# Using Spatial Transcriptomics to Identify a Diagnostic Molecular Signature Associated with Reversible Dental Pulpitis

**DOI:** 10.21203/rs.3.rs-7955718/v1

**Published:** 2026-01-12

**Authors:** Samer Helal Zaky, Dhivyaa Rajasundaram, Alyssa Lu Lee, Kurt Summersgill, Steven Levine, Osama Boulos, Giuseppe Intini, Charles Sfeir, Renato Menezes Silva

**Affiliations:** University of Pittsburgh School of Dental Medicine; Children’s Hospital of Pittsburgh of UPMC; Children’s Hospital of Pittsburgh of UPMC; University of Pittsburgh School of Dental Medicine; University of Pittsburgh School of Dental Medicine; University of Pittsburgh School of Dental Medicine; University of Pittsburgh School of Dental Medicine; University of Pittsburgh School of Dental Medicine; University of Pittsburgh School of Dental Medicine

**Keywords:** Spatial transcriptomics, pulpitis, reversibility profile, fibroblasts, macrophages

## Abstract

**Background:**

Diagnostic protocols in endodontics rely heavily on subjective pain assessments and sensibility testing, which often fail to reflect the true histopathological and molecular state of the dental pulp. This limitation can result in misdiagnosis of reversible pulpal inflammation (pulpitis) as irreversible, leading to unnecessary devitalization of teeth that might otherwise respond to more conservative treatments. Improved understanding of pulp biology is essential to refine case selection and maintain tooth vitality.

**Objective:**

We aim to investigate the transcriptomic profiles of pulpitis, identify inflammation subtypes, and improve classification beyond the traditional reversible/irreversible framework.

**Methods:**

Spatial transcriptomics (Visium-CytAssist-V2) was used to analyze four dental pulp tissues. Cell deconvolution and differential gene expression analyses were performed to characterize transcriptomic signatures of healthy pulp (HL), reversible pulpitis (RP), and irreversible pulpitis (IP), with specific attention to coronal regions adjacent to carious lesions.

**Results:**

Clinically diagnosed IP samples, presenting with deep caries, percussion sensitivity, and lingering cold pain, shared molecular features with RP, including a similar immune-fibroblast ratio and activation of TLR4 and neuroinflammation pathways. IP samples showed upregulation of genes involved in immune signaling, cell migration, and tissue repair. Notably, increased *PTN* and *CXCL14*, along with decreased *ENG (CD105)*, *SELE*, *COL4A1*, *CXCL1*, and *CXCL13*, may indicate residual healing potential. Immune-fibroblast interactions, rather than macrophage shifts alone, appear to influence pulpitis progression.

**Conclusion:**

Our data suggest a distinct transcriptomic profile associated with pulp healing. These findings support a more nuanced diagnostic approach that emphasizes immune-fibroblast dynamics, with implications for improving pulp preservation through appropriate vital pulp therapy.

## INTRODUCTION

The tooth is a mineralized structure with a soft tissue core – or heart: the dental pulp. Traditionally, when dental caries extends through the dentin and reaches the pulp, the standard treatment is root canal therapy (RCT). In this procedure, the inflamed or necrotic pulp tissue is removed and the root canal space is filled with an inert sealing material. More recently, however, Endodontics has shifted toward minimally invasive approaches that aim to preserve pulp vitality (1).

Today, pulp inflammation is classified as either reversible (RP) or irreversible pulpitis (IP), but this distinction is based largely on clinical signs and symptoms rather than a true assessment of the pulp’s healing potential. Current diagnostic methods rely heavily on the subjective experience of pain, which often fails to reflect the actual tissue immunological state (1). For some IP cases, emerging evidence shows that the pulp may retain its immune capacity to shift from a pro-inflammatory to an anti-inflammatory state (1, 2). As a result, many cases labeled as “irreversible” may still retain healing potential, suggesting that more conservative treatments could preserve pulp vitality (3).

A better understanding of the pulp’s actual healing potential would improve diagnosis and allow for more targeted treatments, ultimately preserving pulp vitality that is crucial for extending the lifespan of the tooth.

To overcome current limitations, two key challenges must be addressed. First, pulpitis diagnosis must be based on a molecular classification that reflects true stages or types of inflammation, rather than ambiguous clinical labels (3–5). Second, a non-invasive chairside test is needed to disclose the tissue’s inflammatory profile. While saliva and gingival crevicular fluid show promise, their diagnostic value remains inconclusive (6, 7).

This work is geared to address the first challenge. While bulk RNA sequencing has offered valuable insights into pulpitis (2), advances in single-cell and spatial transcriptomics now make it possible to map the cellular and molecular architecture of pulp tissue in unprecedented detail. Our ongoing study used spatial transcriptomics to characterize the inflammatory events of pulpitis in a higher resolution that could help refining its classification based on the molecular picture of inflammation.

In comparing clinically diagnosed cases of RP and IP, we began with the hypothesis that each condition possesses a distinct cellular profile. This profile encompasses the distribution and proportion of various pulp cell types – including fibroblasts, immune cells, odontoblasts, endothelial cells, and glial cells – as well as differences in their gene expression patterns. Furthermore, we examined the spatial localization of these cells and their transcriptomic signature within the same pulp tissue, particularly in relation to their proximity to the advancing carious lesion.

As we report and discuss a transcriptomic profile potentially indicative for reversibility, the findings of this study underscore the importance of characterizing the cellular and molecular landscape of pulpitis to inform more precise diagnostic and therapeutic strategies. By identifying distinct cellular signatures and spatial arrangements in RP versus IP, we provide valuable insights that may aid in the development of molecular-based criteria for differentiating between reversible and irreversible pulp inflammation, a translational implications that extend well beyond dentistry. This knowledge ultimately supports more conservative treatment approaches that prioritize the preservation of dental pulp vitality, aligning with the broader goal of minimally invasive dentistry and long-term tooth preservation.

## MATERIALS AND METHODS

Data presented in this study come from four dental pulp samples collected from four adult subjects recruited through the University of Pittsburgh School of Dental Medicine’s Emergency and Oral and Craniofacial Surgery clinics, in accordance with IRB protocol #23050001.

### Patient Selection, clinical Evaluation and Sample Collection (Fig. 1).

Upon admission, participants’ medical and pain histories, as well as current medications, were reviewed. To establish pulpal and periapical diagnoses (as determined by the American Association of Endodontists), a standardized intraoral examination was performed, including percussion and palpation tests, along with a cold sensibility test using Endo-Ice Pulp Vitality Refrigerant Spray (Coltene Whaledent, OH, US), to assess response time (immediate vs. delayed) and the duration of lingering pain. Intraoral periapical radiographs were obtained to evaluate the extent and depth of carious lesions and to identify any periapical radiolucencies. At least two additional teeth were tested and used as controls.

Participants from both genders were eligible to participate in the study if they were over 18 years of age, medically healthy, and presented with a vital tooth without radiographic signs of periapical pathosis.

Participants were excluded from the study if they required antibiotic prophylaxis; used tobacco or alcohol; were pregnant; had diabetes or blood disorders; had recently used anti-inflammatory drugs, antibiotics, or antidepressants; or showed signs of poor oral hygiene, such as heavy plaque, severe gingivitis, or periodontitis.

Patients with irreversible pulpitis were invited to take part only after root canal treatment was clearly recommended as the best option. Tooth extraction was done only if the tooth couldn’t be saved or if the patient chose it for financial or personal reasons.

All participants were informed about the study’s purpose and procedures and provided written informed consent prior to enrollment.

### Details of the samples included in the study (Fig. 1).

#HL1: Healthy pulp from an extracted upper right third molar of a 31-year-old male. #RP1: Pulp from an upper left third molar with caries and reversible pulpitis (RP), extracted from a 42-year-old male; the opposing tooth was missing. #IP1: Diagnosed with symptomatic irreversible pulpitis (IP); pulp collected from the lower right first molar of a 21-year-old female. #IP2: Diagnosed with symptomatic irreversible pulpitis; pulp collected from the lower left second molar of an 18-year-old male.

### Tissue Collection and Processing.

Immediately after extraction, teeth were decoronated using a high-speed drill. A stainless-steel insect pin (Austerlitz, Ø 0.1 mm) was inserted into the pulp chamber to mark tissue orientation, distinguishing regions adjacent to the carious lesion from those farther away. Pulp tissue was then carefully extirpated as intact as possible and fixed in 10% neutral buffered formalin for 24 hours.

Tissue samples were subsequently processed for paraffin embedding and histological sectioning. Embedding was performed in stainless steel base molds (Epredia^™^, 24 × 24 × 5 mm^3^, Fisher Scientific) with a marked 6 × 6 mm square to ensure proper fit on barcoded spatial transcriptomics slides. Sample was sectioned in 5μm-thick sections for histopathological analysis with H&E stain and for detection of bacterial cells within the pulp tissue with Brown & Brenn stain (Electron Microscopy Sciences #26105). Following RNA quality control (10μm sections), samples were subjected to spatial transcriptomics and lineage tracing analyses using the Visium CytAssist V2 platform (10x Genomics^™^) at the Genetic Sequencing Core Facility of UPMC Children’s Hospital of Pittsburgh.

### Spatial Transcriptomics Data Integration and Analysis.

Spatial transcriptomics data were generated from four tissue sections representing distinct sample conditions: #HL1, healthy pulp from 3rd molar extraction; #RP1, clinically diagnosed reversible; #IP1 and #IP2 both clinically diagnosed irreversible pulpitis. Each tissue section was processed using the Visium Spatial Gene Expression platform, producing spatially resolved gene expression count matrices corresponding to defined spots across the tissue.

Raw count data from each section were initially preprocessed independently. This included filtering out low-quality spots and genes expressed at very low levels to reduce noise and improve downstream analyses. Each dataset was then normalized separately using the normalization method implemented in Seurat v5, which corrects differences in sequencing depth across spots by scaling gene expression counts to a fixed total count per spot followed by log-transformation.

To integrate the four datasets and align shared biological signals while minimizing technical batch effects inherent to separate tissue preparations, canonical correlation analysis (CCA) was employed using Seurat’s integration workflow. Anchors between datasets were identified, enabling harmonization of the data into a single integrated Seurat object. This integrative approach allowed robust cross-sample comparison of spatial gene expression patterns while preserving spatial context within each tissue section.

### Cell Type Deconvolution.

To annotate spatial spots with cell type identities, RCTD (Robust Cell Type Decomposition) was performed using reference single-cell RNA-seq data from Krivanek et al (8). This approach enabled the identification of ten major cell types across samples: Endothelial cells, Glial cells, Immune cells, Macrophages, Odontoblasts, Peri-odontoblasts, Perivascular cells, Pulp fibroblasts, Pulp fibroblasts from the apical papilla (AP), and Pulp fibroblasts with periodontal ligament (PDL) characteristics. Deconvolution results were used for downstream spatial mapping and cell type–specific analyses.

### Differential Expression Analysis.

Differential gene expression (DEG) analyses were conducted comparing entire tissue sections and selected ROIs to identify genes and pathways associated with disease states and spatial microenvironments. Spatial feature volcano plots were generated to visualize gene expression patterns within tissue context. We performed DEG among and within samples. The analysis among the four samples was on three comparison levels: pulpitis (RP and IP) vs. healthy, IP (#IP1 and #IP2) vs. RP (#RP1) and between the two IP samples #IP1 vs. #IP2. The analysis within each sample considered a region of interest (ROI) facing the carious lesion vs. in the pulp chamber as a whole.

### Region of Interest (ROI) Selection.

Regions of interest (ROIs) were manually selected using the CellSelector. Selection criteria were based on histological features and spatial proximity to the carious lesion, focusing on the pulp chamber area most directly impacted by caries invasion. This manual annotation allowed targeted investigation of transcriptional changes within microenvironments affected by disease progression.

Module scores representing pathway activity (e.g., inflammatory response, neuroinflammatory response, TLR4 signaling) were calculated using Seurat’s AddModuleScore function and visualized as spatial feature plots and violin plots to assess spatial and cell type–specific pathway activation. Statistical comparisons were performed as appropriate to determine significance.

### Visualization and Software.

All analyses and figure generation were performed using R (version 4.4) with Seurat v5, RCTD, and visualization packages such as ggplot2.

### Protein Expression Data.

Protein expression (PEX) data from Visium CytAssist technology were integrated and visualized as spatial feature plots to correlate transcriptomic findings with protein-level expression patterns, particularly focusing on markers relevant to inflammation and pulp pathology.

### Statistical analysis.

Raw gene expression data were first quality-controlled and normalized using Seurat’s default SCTransform-based normalization workflow, which corrects for technical variation and ensures comparability across samples. Differential expression analysis between conditions (e.g., healthy, RP, IP) and regions (e.g., facing vs. away from the carious lesion) was performed using Seurat’s v5 function to calculate average expression scores for gene sets of interest (e.g., immune activation, fibroblast remodeling pathways) within each spatial spot. Statistical comparisons were made using Wilcoxon rank-sum tests, with significance defined as with |log2 fold-change| > 2 and an adjusted p-value < 0.05 after Bonferroni correction for multiple testing. All analyses were performed to visualize spot-level expression patterns overlaid on histological images, enabling spatial interpretation of transcriptional differences.

## RESULTS

### Histopathological analysis.

This study analyzed four human dental pulp samples using both histopathology and spatial transcriptomics. Sample #HL1 was a healthy pulp from a third molar impaction surgery, #RP1 was clinically diagnosed as reversible pulpitis, and #IP1 and #IP2 were diagnosed as irreversible pulpitis.

**Figure 1:**
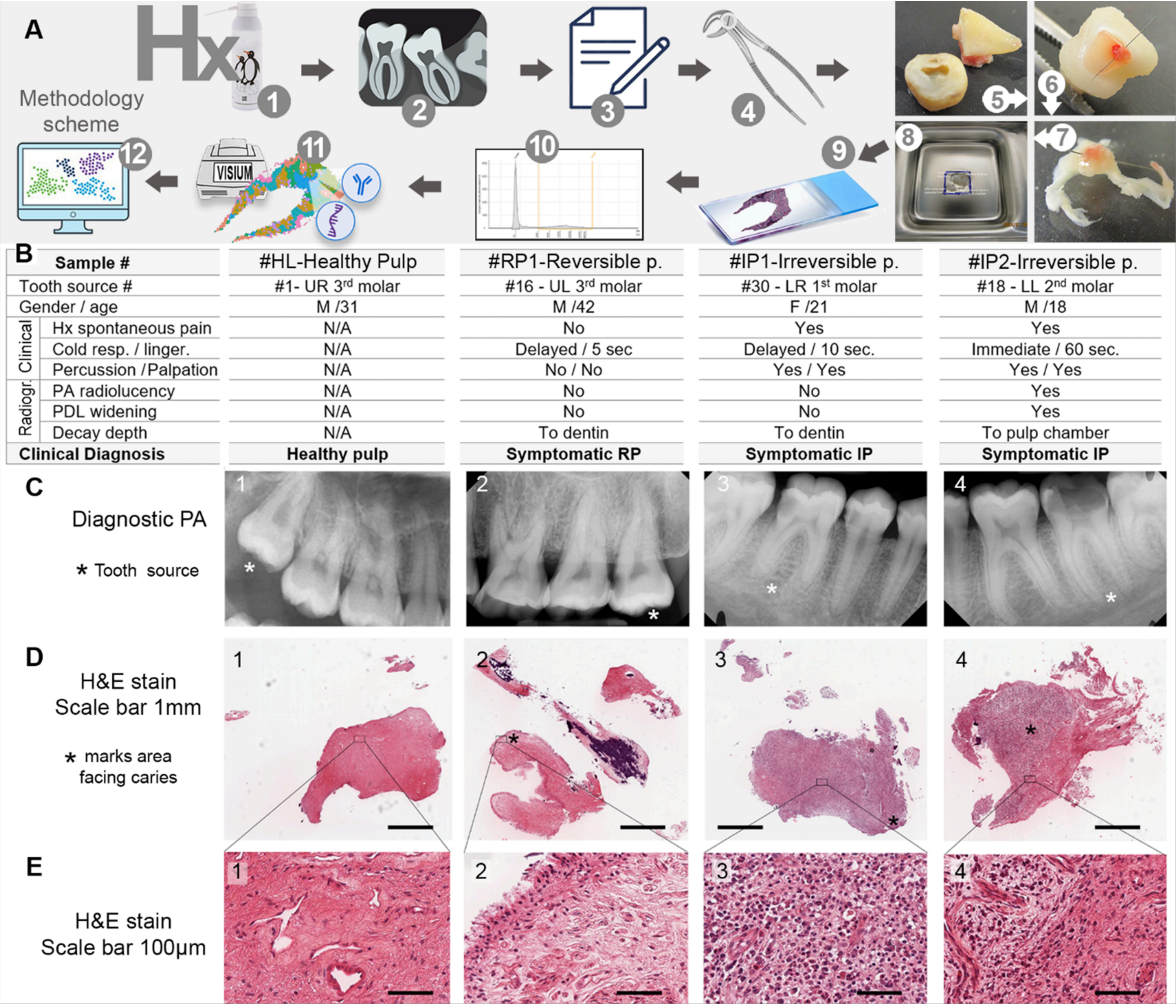
Methodology, Clinical diagnosis, and histological analysis of the 4 samples. **A:** Experiential design and Methodology: 1- History and intraoral examination for clinical diagnosis of pulpitis. 2- Periapical radiograph. 3- Patient qualification and consent form. 4-Tooth extraction. 5- Tooth decoronation. 6-Marking the pulp tissue facing the carious lesion. (Notably, some samples revealed a localized area of inflammation that would typically qualify the tooth for direct pulp caping or vital pulp therapy (VPT) rather than RCT or extraction.) 7- Pulp extirpation. 8- Formalin fixation and 6.5×6.5mm histology processing. 9- H&E stain for histopathological analysis and Brown & Brenn stain for checking the presence of bacterial cells within the pulp indicating a pulpal exposure by the caries. 10- RNA quality control by % of RNA fragments > 200 nucleotides (DV200). 11-Sample processing for Spatial transcriptomics (Visium CytAssist V2) for total transcriptome and selected inflammatory proteins. 12-Spatial transcriptomics data analysis and visualization. **B:** Data from clinical examination and clinical diagnosis for the 4 included samples #HL1: healthy pulp; #RP1: Reversible pulpitis; #IP1 and #IP2: irreversible pulpitis. N/A in HL indicated no clinical assessment in extracted impacted 3^rd^ molars. **C:** Periapical radiograph of respective samples (C1–4); * points to the tooth source. **D:** H&E with scale bars 1mm for respective samples (D1–4); * marks area facing the carious lesion. **E:** H&E high magnification from insert in D of respective samples (E1–4; scalebars 100μm). D&E show samples #HL1 and #RP1 primarily occupied by fibrovascular tissue, #IP1 predominantly populated by inflammatory cells while #IP2 shows distinct two zones: inflammatory adjacent to the carious lesion and fibrotic away from the carious lesion.

Histopathologically, the #HL1 sample, displayed fibrovascular tissue with no signs of edema or inflammation. Pulp fibroblast nuclei and blood vessels were evenly distributed throughout (Fig. 1D1 & E1). In contrast, #RP1 showed more edematous fibrovascular tissue than the healthy sample but still lacking inflammatory cells. The peripheral tissue retains an intact layer of odontoblasts (Fig. 1D2 & E2).

#IP1 and #IP2 (Fig. 1B) were intensely inflamed, but with difference inflammatory cells populations and distribution. The tissues in #IP1 were severely inflamed with acute response evident by a uniform distribution of neutrophils (Fig. 1D3&E3). Areas of chronic response show edema with plasma cells and lymphocytes. The area at the caries front (asterisk in Fig. 1D3) appears to be less inflamed, but with increased vascularity. #IP2, in contrast, presented with typical clinical symptoms (Fig. 1B) and radiographic features (Fig. 1C4) consistent with IP but its histopathological analysis revealed a different picture. #IP2 displayed two distinct zones: one near the caries front (asterisk in Fig. 1D4) with localized chronic inflammation dominated by plasma cells and lymphocytes without neutrophils, and a separate region of healthy fibrovascular tissue free of inflammatory cells (Fig. 1D4&E4). This fibrous zone, different from pulp fibrosis, was highly cellular and richly vascularized (Fig. 1E4).

Caries excavation post-extraction and Brown and Brenn staining (data not shown) confirmed that none of the samples had pulp exposure or detectable bacterial cells in the pulp tissue.

### Spatial Transcriptomic clustering and cell type deconvolution

**Figure 2:**
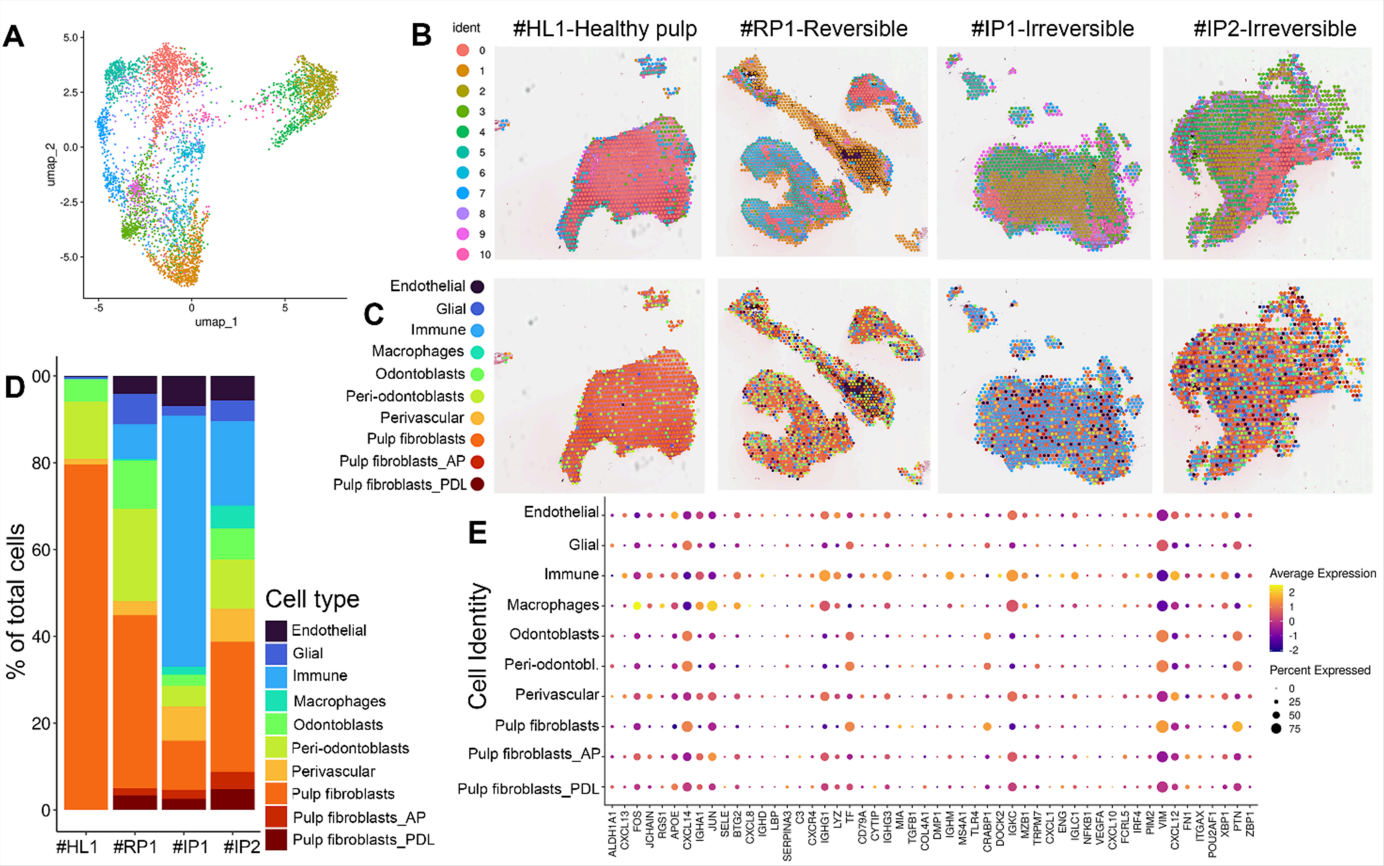
Spatial Transcriptomic clustering and cell types. (A) Uniform Manifold Approximation and Projection (UMAP) of integrated spatial transcriptomic data, where each dot represents a spot and is colored by its assigned cluster. (B) Spatial localization of these clusters across tissue sections, grouped by condition (healthy pulp, reversible pulpitis, and irreversible pulpitis), illustrating variation in gene expression patterns within and between conditions. (C) Cell type deconvolution of spatial transcriptomics data from four tissue samples, based on reference profiles from Krivanek et al. (8), identifying ten major cell types: endothelial cells, glial cells, immune cells, macrophages, odontoblasts, peri-odontoblasts, perivascular cells, pulp fibroblasts, pulp fibroblasts from apical papilla (AP), and pulp fibroblasts from periodontal ligament (PDL). (D) Bar plots showing the proportion of each deconvoluted cell type across the four samples. Samples #RP1 (reversible pulpitis) and #IP2 (irreversible pulpitis) exhibit similar immune-to-fibroblast (I/F) ratios, while #IP1 (irreversible pulpitis) shows a higher I/F ratio typical of this condition. The healthy control (#HL1) shows a low I/F ratio, characteristic of normal tissue. Notably, fibroblastic populations from AP and PDL are more abundant in pulpitis samples. (E) Dot plot of selected marker genes across deconvoluted cell types. Dot size represents the percentage of spots expressing the gene within each cell type, and color intensity reflects average expression. This visualization highlights distinct transcriptional signatures among cell types and reveals subtle differences between fibroblast subpopulations.

We integrated spatial data from multiple sections/samples using canonical correlation analysis (CCA) and performed unsupervised clustering with a graph-based approach, identifying ten distinct clusters with a uniform distribution of unique transcripts (Fig. 2A). To contextualize these clusters and evaluate their spatial arrangement, we projected the spots onto the brightfield image of the corresponding tissue section stained with hematoxylin and eosin (H&E) in Fig. 2B.

Deconvolution analysis of the 4 samples identified the presence of ten major cell types: Endothelial cells, Glial cells, Immune cells, Macrophages, Odontoblasts, Peri-odontoblasts, Perivascular cells, Pulp fibroblasts, Pulp fibroblasts from the apical papilla (AP), and Pulp fibroblasts from periodontal ligament (PDL) (Fig. 2C&D). The spatial profiling (Fig. 2C) and the percent of total cells in each sample presented by bar plots (Fig. 2D) revealed a pattern of cell clustering in #IP2 similar to #RP1 (RP), including a similarly low immune-to-fibroblast (I/F) ratio. In contrast, consistent with irreversible inflammation, the #IP1 demonstrated both histologically and transcriptomically a high I/F ratio, marked by a dominant immune cell presence and reduced fibroblast content. As expected, the healthy control (#HL1) showed a low I/F ratio with strong fibroblast predominance.

Interestingly, while that 80% predominant population in the healthy sample (#HL1) was exclusively Pulp fibroblasts, expressing CXCL14, TF, CRABP1, VIM, PTN among others, the three pulpitis samples (RP and IP) showed infiltration of other fibroblastic populations characterized as fibroblasts from AP (or with AP characteristics) or fibroblasts from the PDL (or with PDL characteristics) expressing less CXCL14 and more CXCR4 (Fig. 2E). These findings may suggest that during pulpitis different cells populations traffic from outside to within the dental pulp (or from the radicular pulp to the pulp chamber) contributing towards a certain pulpitis profile.

Because this study analyzed extirpated pulps, damage to the odontoblastic layer is expected. We hence primary focused on characterizing the immune and fibroblasts populations.

**Figure 3:**
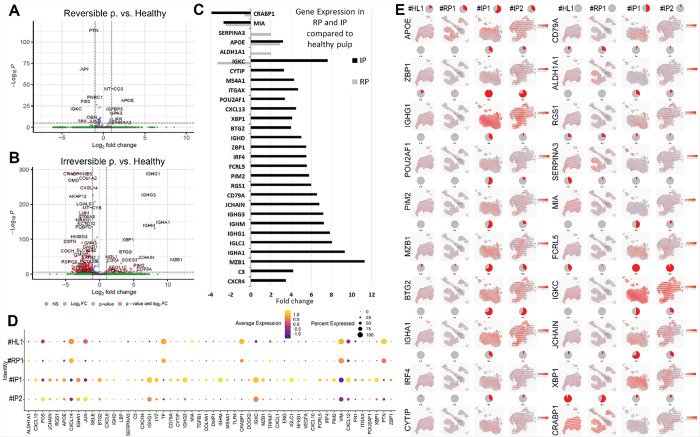
Comparing Reversible and Irreversible pulpitis to healthy pulp. A: The volcano plot displays transcriptomic differences between reversible pulpitis and healthy pulp tissue. Each point represents a gene. The x-axis shows the log_2_ fold change (log2FC), with positive values indicating genes upregulated in reversible pulpitis and negative values indicating genes upregulated in healthy pulp. The y-axis shows the −log_10_ of the adjusted p-value, representing the statistical significance of the differential expression. Genes that meet the fold change cutoff and strong statistical significance appear in red. Threshold lines indicate the significance cutoff (e.g., adjusted p-value < 0.05) and fold change threshold (e.g., |log_2_FC| > 0.25). **B:** Volcano plot of transcriptomic profile for irreversible pulpitis versus healthy pulp. **C:** Bar graph summary of top regulated genes from A and B with highest fold change in reversible and irreversible pulpitis compared to healthy pulp expressed in > 2-fold change with adjusted *p* values <0.05. **D:** Dot plot showing average expression and proportion of expressing spots for selected genes across the four sections. Each dot represents a selected gene’s expression within a given cell type. The size of the dot indicates the percentage of spatial spots (within that cell type) expressing the gene, while the color intensity reflects the average expression level. **E:** Spatial feature plots display the expression of selected genes (with highest fold change) across the four tissue samples. In each plot, gene expression is overlaid on the tissue sections, where color intensity reflects expression levels at each spatial spot. For each gene, a pie chart summarizes the proportion of total expression contributed by each sample, allowing for comparison across conditions or tissue states.

**Figure 4:**
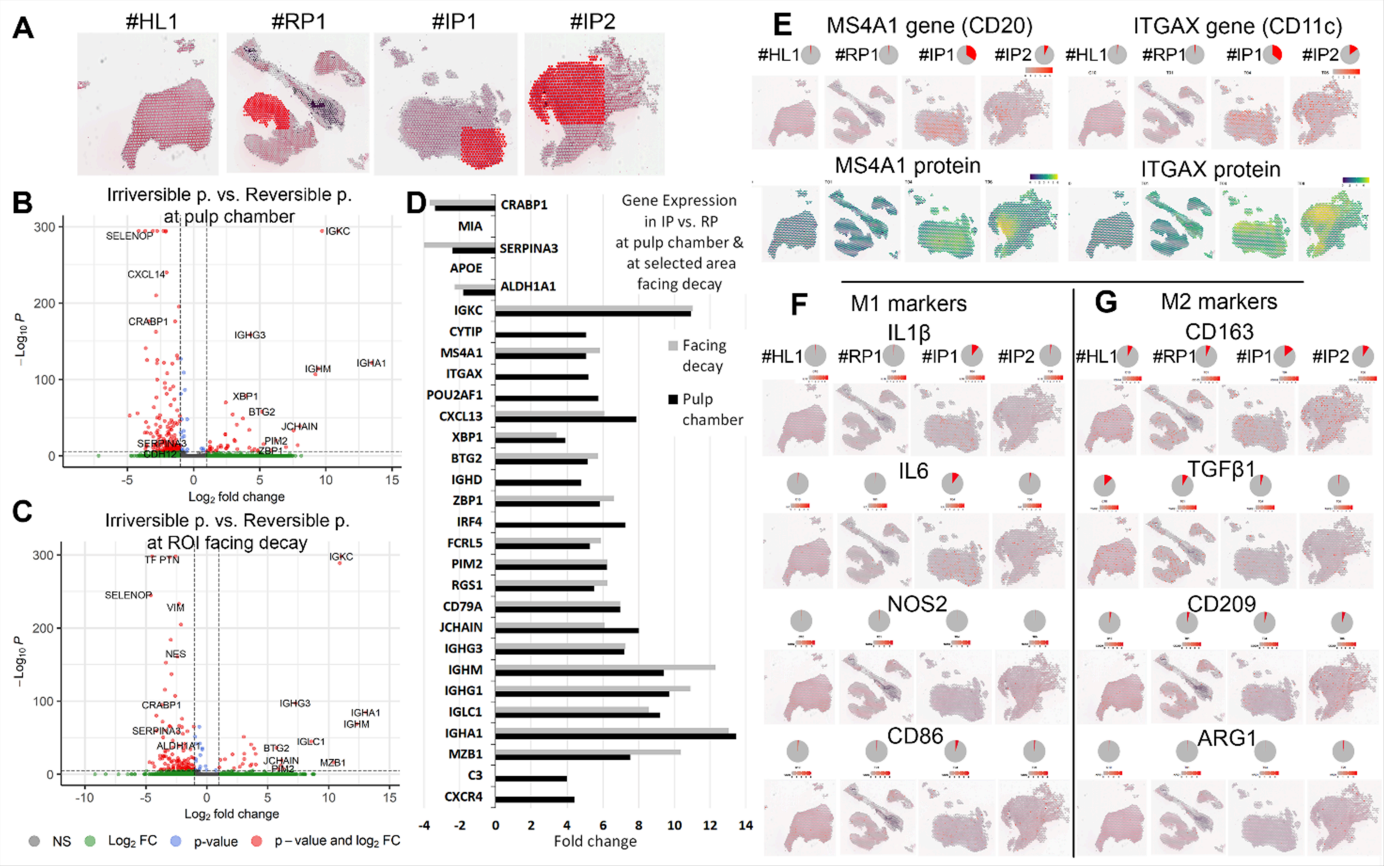
Comparing Reversible versus Irreversible pulpitis. A: ST maps for the four samples with annotated ROIs outlining the pulp chamber area facing the invading carious lesion. ROIs were manually selected based on histological context and spatial proximity. Highlighting these specific zones allows for focused analysis of transcriptional changes within the tissue microenvironment that is most directly impacted by caries progression. **B:** Volcano plot of transcriptomic profile for IP versus RP in the whole pulp chamber. X-axis: log2FC; Y-axis–log_10_ of adjusted p-value, representing the statistical significance of the differential expression. Genes that meet the fold change cutoff and strong statistical significance appear in red. Threshold lines indicate the significance cutoff (e.g., adjusted p-value < 0.05) and fold change threshold (e.g., |log2FC| > 0.25) **C:**Volcano plot of transcriptomic profile for IP versus RP in the ROI facing the caries. **D:** Bar graph summary of top regulated genes from B and C with highest fold change in IP versus RP comparing the whole pulp chamber with the area facing the caries expressed in > 2-fold change with *p*values<0.05. **E:** Spatial feature plots display the expression of MS4A1 and ITGAX transcripts across the tissue sections, generated using Visium CytAssist ST technology. Color intensity indicates expression levels at each spatial spot. Corresponding protein expression (PEX) data for MS4A1 and ITGAX are shown alongside, revealing co-localization and confirming transcriptomic findings at the protein level. Pie charts summarize the proportion of total gene and protein expression. **F:** Spatial feature plots display the expression levels of canonical M1 macrophage markers (IL1β, IL6, NOS2, CD86). Color intensity reflects the spatial localization and relative abundance of gene expression at each spot. Pie charts summarize the proportion of total expression for each gen. **G:** Spatial feature plots show the distribution and expression levels of markers associated with M2-like (anti-inflammatory) macrophages (CD163, TGFβ1, CD209, ARG1).

**Figure 5:**
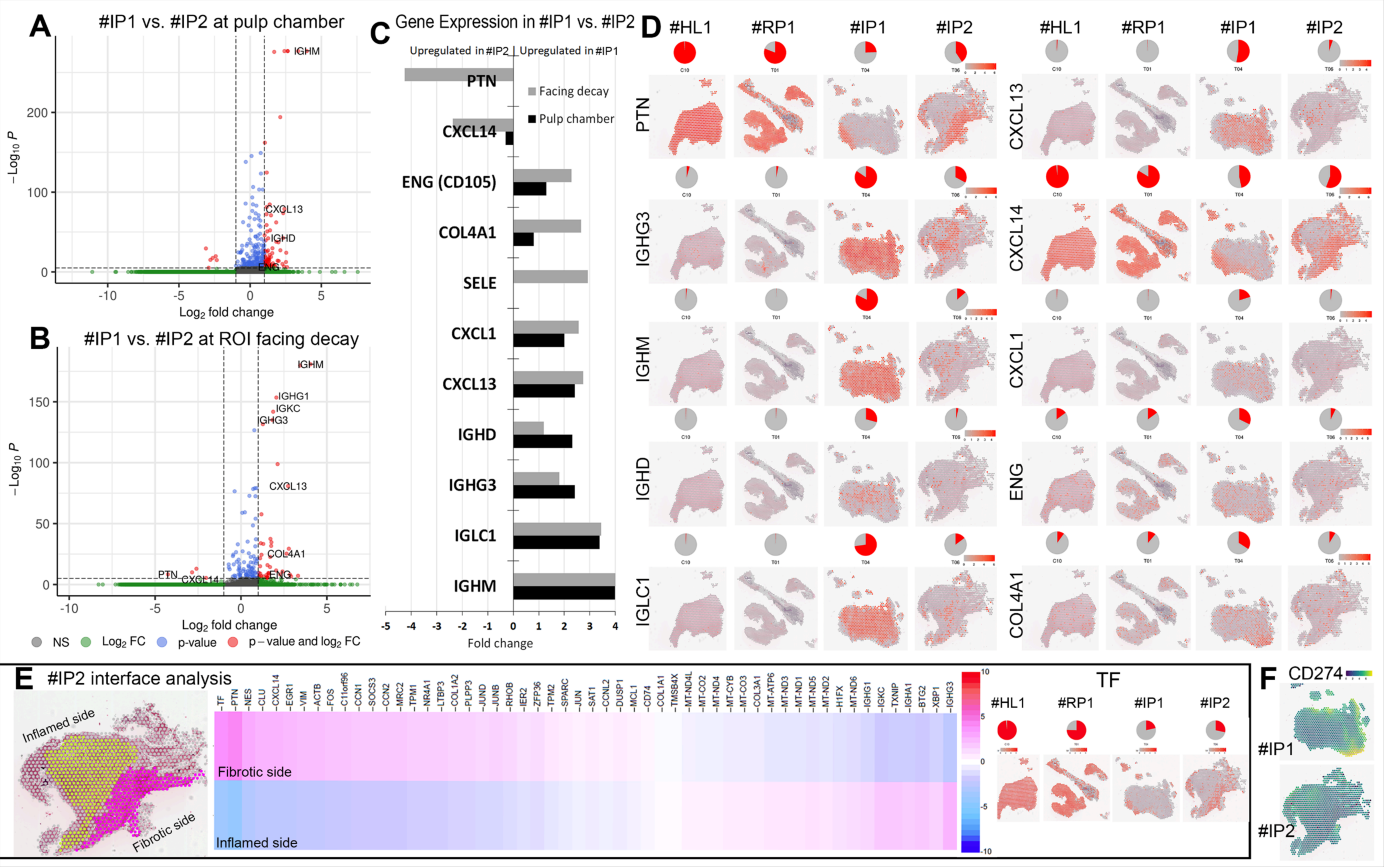
Comparing the two irreversible pulpitis samples #IP1 and #IP2. A: Volcano plot of transcriptomic profile #IP1 versus #IP2 in the whole pulp chamber. The x-axis: log2FC; Y-axis −log_10_ of the adjusted p-value, representing the statistical significance of the differential expression. Genes that meet the fold change cutoff and strong statistical significance appear in red. Threshold lines indicate the significance cutoff (e.g., adjusted p-value< 0.05) and fold change threshold (e.g., |log2FC| > 0.25) **B:** Volcano plot of transcriptomic profile #IP1 versus #IP2 in the ROI facing the caries. **C:** Bar graph summary of top regulated genes from B and C with highest fold change in the clinically diagnosed IP sample #IP1 vs. the other clinically diagnosed IP sample #IP2 in the whole pulp chamber and limited to the area facing the caries expressed in>2-fold change with *p*values<0.05. The analysis revealed PTN and CXCL14 upregulated in #IP2 and IGHD, IGHG3, IGLC1, IGHM, CXCL13 ENG, COL4A1, SELE, CXCL1 upregulated in #IP1 with the latter four markers particularly enriched at area facing the caries. **D:**Spatial feature plots illustrate the expression patterns of selected genes across the four tissue samples, highlighting the similarity between #IP2 and #RP1. Each plot overlays gene expression intensity on the tissue section, where color brightness reflects the level of expression at each spatial spot. **E:**Comparison of gene expression in #IP2 between its two histologically (Fig.1 E4&D4) and transcriptomically (Fig.2B) distinct areas: Inflamed side facing the caries (yellow selection) and fibrotic side (pink selection). The heatmap of differential gene expression of the inflamed side vs. the fibrotic side show upregulation of PTN and TF in the fibrotic side and upregulation of IGHG3 on the inflamed side. **F:** Spatial feature plots show CD274 protein overexpression specifically in the region facing carious invasion within sample #IP1 indicating a localized overexpression in response to bacterial endotoxins from active caries invasion.

To gain better insights into how individual cell-types responded to pulpitis we performed differential gene expression (DEG) analysis among and within samples. The analysis among the four samples was on three comparison levels: pulpitis (RP and IP) vs. healthy (Fig. 3), IP (#IP1 and #IP2) vs. RP (#RP1) (Fig. 4) and between the two IP samples #IP1 vs. #IP2 (Fig. 5). The analysis within each sample considered a region of interest (ROI) facing the carious lesion versus in the pulp chamber as a whole.

### Comparing Reversible and Irreversible pulpitis to healthy pulp

Differentially expressed genes (DEGs), visualized in volcano plots (Fig. 3A–B) and summarized in a bar graph of top regulated genes (fold change > 2, adjusted p < 0.05; Fig. 3C), were confirmed by spatial feature plots (Fig. 3E) and dot plots (Fig. 3D). Most upregulation occurred in samples with advanced caries (#IP1, #IP2), while expression in the RP sample (#RP1) resembled that of healthy pulp (#HL1).

Most of the upregulated genes in IP were immunoglobulin-related (e.g., *IGHD, IGHG3, IGHM, IGHG1, IGHA1, JCHAIN, MZB1*) primarily expressed by B cells and plasma cells. Additional upregulation was seen in C3-activated fibroblasts (9) and in shared immune–fibroblast markers such as *CXCL13* and *CXCR4*.

In the comparison to healthy pulp, genes such as *ZBP1, POU2AF1, PIM2, MZB1, BTG2, IRF4, CYTIP, CD79A, RGS1, FCRL5*, and *XBP1* were highly expressed in sample #IP1, but showed lower expression in #IP2 and #RP1. This pattern suggests their potential use as markers for identifying reversible pulpitis.

Apolipoprotein E (APOE), known to suppress macrophage proinflammatory responses to lipopolysaccharide (10), was upregulated in both RP and IP samples, showing comparable expression levels in #RP1 (RP) and #IP2 (IP). *APOE* is reported to be expressed in healthy adult pulp (8) and upregulated from odontoblasts in cases presenting deep caries lesions (11).

SERPINA3 and ALDH1A1 were upregulated only in RP. SERPINA3, a protease inhibitor expressed by some odontoblasts and glial cells, protects tissues from neutrophil-mediated damage during deep caries (10, 11). ALDH1A1, a stemness and odontoblast differentiation marker (12, 13), likely reflects early inflammatory responses, showing localized expression in early caries (#RP1) and patchy presence in advanced cases (#IP1, #IP2) without altering overall sample levels.

MIA, a fibroblast adhesion molecule, was equally downregulated in both RP and IP. In contrast, CRABP1, which regulates retinoic acid synthesis and supports overall tissue homeostasis (14), showed progressive downregulation with increasing caries severity.

Notably, Immunoglobulin Kappa Constant (IGKC), expressed by plasma cells, was downregulated in RP, and upregulated in IP compared to healthy pulp. Samples #IP1 and #IP2 showed high IGKC expression; however, additional markers (Fig. 5) suggest that #IP2 aligns more closely with a reversible profile. This indicates that IGKC may reflect the extent or spread of caries rather than the severity of inflammation that determines pulpitis reversibility.

### Comparing Reversible versus Irreversible pulpitis (Fig. 4).

We compared the two IP samples (#IP1 and #IP2) to the RP sample (#RP1), focusing on both the entire pulp chamber and the ROI facing the carious lesion (Fig. 4A). Differential expression analysis using volcano plots (Fig. 4B&C) and a bar graph summary (Fig. 4D) showed that CRABP, SERPINA3, and ALDH1A1 were upregulated in RP, with no enrichment near the caries site. In contrast, BTG2, ZBP1, FCRL5, RGS1, IGHM, IGHG1, and MZB1 were elevated in IP, especially at the site of the caries invasion.

Membrane Spanning 4-Domains A1 (MS4A or CD20), a surface marker involved in B cell activation and proliferation (15) and expressed in irreversible pulpitis (16), was more highly expressed near the area adjacent to the carious lesion. Similarly, Integrin Subunit Alpha X (ITGAX or CD11c), which plays a key role in immune cell interactions and is actively involved in pulpitis (17, 18), showed broader expression across the pulp chamber, with higher protein levels concentrated near the site of caries invasion in sample #IP2.

Both genes were previously reported as upregulated in RP and deep caries (10, 19). Their spatial feature plots and pie charts revealed higher expression in IP compared to RP, and in sample #IP1 versus #IP2. The respective protein expression (PEX) showed localized areas of high intensity in #IP2 (Fig. 4E), confirming that events beyond transcription are significantly influencing the final amount of functional protein in the cell.

### Comparing the two irreversible pulpitis samples #IP1 and #IP2.

Noting the distinct transcriptomic profile of the two samples clinically diagnosed as IP, we performed a comparative analysis between #IP1 and #IP2.

Differential expression analysis (Fig. 5A–C) revealed that Pleiotrophin (PTN) and CXCL14 were upregulated in sample #IP2, making its transcriptomic profile similar to that of the RP sample (#RP1), despite being clinically diagnosed as IP. In contrast, other genes, endoglin (*ENG or CD105*), COL4A1, selectin E (SELE), IGHD, IGHG3, IGLC1, IGHM and the proinflammatory cytokines CXCL1, CXCL13 were upregulated in #IP1 only. ENG, COL4A1, SELE and CXCL1 were particularly enriched at area facing the carious lesion. These findings show that the transcriptomic profile of the clinically diagnosed IP sample #IP2 resembled that of the RP (#RP1) sample; suggesting the presence of potential markers for predicting pulpal healing capacity and confirming the notion that some clinically diagnosed IP cases do, at the transcriptomal level, retain features of reversibility (4).

Histopathological analysis showed that sample #IP1 had widespread inflammatory cell infiltration, while sample #IP2 displayed two distinct regions: an inflamed side and a fibrotic side (Fig. 1D3, Fig. 2C–D). We analyzed the immune–fibroblast interface to identify markers that may localize inflammation and predict the potential for pulpitis reversal. Differential expression analysis, illustrated by the heatmap (Fig. 5E) and spatial feature plots (Fig. 5D–E), revealed upregulation of Pleiotrophin (PTN) and Transferrin (TF) in the fibrotic region, and IGHG3 in the inflamed region. When comparing #IP1 and #IP2, TF showed minimal fold change, while PTN and IGHG3 were more prominently differentially expressed. This suggests that the regulation of PTN (by fibroblasts) and IGHG3 (by immune cells) may be key in determining the trajectory of pulpitis resolution or progression.

Protein expression (PEX) analysis comparing samples #IP1 and #IP2 revealed localized overexpression of CD274 (PD-L1) in the area of caries invasion in #IP1 only, though the adjusted p-value was not significant (Fig. 5F). CD274, an immune checkpoint protein, inhibits T-cell activation and cytokine release by binding to its receptor (20). While previous studies have shown its role in the immunomodulatory response of dental pulp stem cells (DPSCs) to inflammation (21), this is the first report demonstrating CD274 localized overexpression in pulp tissue possibly in response to bacterial endotoxins from active caries.

Taken together, these findings suggest that although #IP2 showed clinical features consistent with irreversible pulpitis, deep caries reaching the chamber, immediate and prolonged cold sensitivity, it exhibited a molecular inflammatory profile more characteristic of reversible pulpitis.

### Inflammatory pathways analysis

**Figure 6:**
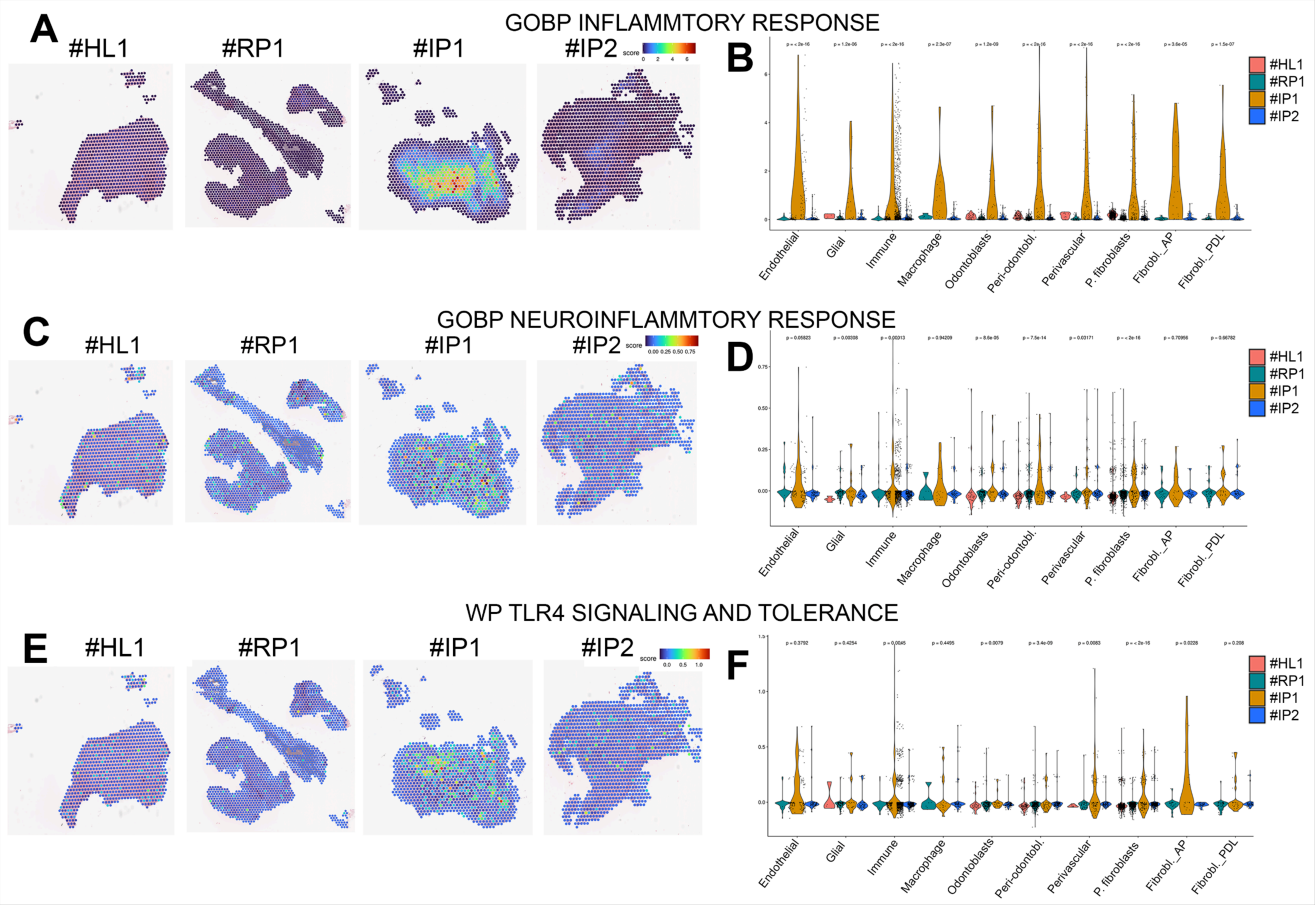
**Module scores representing pathway activities** were calculated for Inflammatory Response (A & B), Neuroinflammatory Response (C & D), and TLR4 Signaling and Tolerance (E & F). (A, C, E) Spatial feature plots show the distribution and intensity of pathway activity across the four tissue sections, highlighting regions of increased pathway engagement. Color intensity corresponds to the relative activity level at each spatial spot. (B, D, F) Violin plots display the distribution of pathway activity scores across different cell types within each sample, revealing cell type–specific variations in pathway activation.

Module scores representing pathway activities for Inflammatory Response (Fig. 6A & B), Neuroinflammatory Response (Fig. 6C & D), and TLR4 Signaling and Tolerance (Fig. 6E & F) show similar patterns in #IP2 and #RP1. Violin plots confirm similar low pathway activation in both, while #IP1 showing strong activation across immune cells, macrophages, endothelial cells, and notably, fibroblasts apical papilla (AP).

## DISCUSSION

Aiming to identify markers of reversibility, this study, first to our knowledge, used spatial transcriptomics to map the cellular and molecular profiles of human dental pulps with different degrees of inflammation. Our findings highlight the dynamic immune-fibroblast interactions and suggest molecular signatures linked to healing potential.

In this study, we chose the American Association of Endodontists (AAE) classification of reversible and irreversible pulpitis over Wolter’s mild/moderate/severe system (4) because it more directly guides treatment decisions: namely, whether to preserve pulp vitality or proceed with devitalization. However, we acknowledge that this binary approach can oversimplify a condition that is often subjective, relying on both patient-reported symptoms and clinical judgment. In the light of emerging research suggesting the dental pulp is more resilient than once believed, the limitations of current diagnostic labels become clear (1, 3, 22).

In comparison to healthy pulp (#HL1), our analysis included one clinically diagnosed RP sample (#RP1) and two IP samples (#IP1 and #IP2). Although #IP2 clinically presented with typical IP signs, deep caries, percussion sensitivity, and lingering pain to cold, it shared molecular features with the RP sample, including a similar immune-fibroblast (I/F) ratio and comparable TLR4 and neuroinflammation pathway activity. In contrast, #IP1 showed extensive immune infiltration and a strongly proinflammatory transcriptomic profile, suggesting a more advanced or aggressive inflammatory state. These findings raise the possibility that #IP1 and #IP2 represent distinct inflammatory responses rather than sequential stages of inflammation (5). In fact, #IP1 exhibited a clearer irreversible profile than #IP2, despite both being labeled under the same clinical diagnosis.

Histologically, #IP2 revealed two distinct regions: an inflamed zone directly beneath the carious lesion, and an adjacent fibrous, vascularized area with no immune infiltration, suggesting a transitional or partially reversible inflammatory state. Spatial transcriptomics analysis of the two zones showed that Pleiotrophin (PTN) and transferrin (TF) were enriched in the fibrotic zone, while IGHG3 was localized to the inflamed region, suggesting a captured interplay between regenerative and inflammatory activity to balance homeostasis. PTN, linked to repair and neuroimmune regulation, may signal resolution from fibroblasts (23, 24), while IGHG3, from Plasma cells or B1a cells subset, reflects ongoing inflammation. These findings confirm the mismatch between subjective clinical symptoms and true tissue state and support prior reports of mixed histological profiles within a single pulp, further complicating the matter of diagnosis (3, 4, 19).

Pain remains an unresolved diagnostic challenge in dental pulpitis, as this study highlights its poor correlation with underlying inflammation. Notably, patient #IP2 reported more severe pain despite displaying a transcriptomic profile closer to reversible pulpitis. Pain in both #IP1 and #IP2 could have resulted from edema-induced pressure on A-delta or C fibers rather than active inflammation (25). We did not observe significant differences in classic pain-related genes (AMELX, CD14, MMP13), but CD79A, previously reported to be downregulated in pain (26), was upregulated. Although #IP1 had abundant neutrophils capable of releasing pro-inflammatory cytokines, this did not align with greater pain symptoms. Instead, #IP2, with less histological inflammation, exhibited more intense pain and a radiographically larger cavity. These findings backing the disconnect between clinical symptoms and molecular pathology underscore the limitations of current subjective diagnostic tools. While we did not assess the contribution of the glial population (Schwann cells) in pain perception, we confirm their participation in the immune response via CXCL14 expression and their contribution to pulpal homeostasis through TF and PTN expression (23, 24).

One key finding in our study is the critical role of immune-fibroblast interactions in shaping dental pulp inflammation. While both #IP1 and #IP2 showed immune infiltration, only #IP1 had a high immune-to-fibroblast (I/F) ratio. In contrast, fibroblasts in #RP1 and #IP2, both with comparatively low I/F ratio, expressed anti-inflammatory and regenerative genes such as CRABP1, SERPINA3, ALDH1A1, PTN, and CXCL14, linked to tissue homeostasis and early immune modulation (8, 10, 23, 24). Meanwhile, #IP1 was enriched in pro-inflammatory and remodeling genes, including ENG (CD105), SELE, COL4A1, CXCL1, and CXCL13, markers of endothelial activation, immune cell recruitment, and matrix remodeling (26–28). These genes may represent a molecular signature of irreversible inflammation, while the presence of PTN and CXCL14 in #IP2 suggests a potential for recovery.

In the dental pulp, CXCR4 is crucial for coordinating immune responses, regulating cell migration, and maintaining tissue homeostasis or facilitating repair (29, 30). The expression of CXCR4 from immune-like fibroblasts in irreversible pulpitis suggests that these fibroblasts actively recruit immune cells and perpetuate chronic inflammation, thereby contributing to tissue damage and the progression of the disease.

These spatial transcriptomic profiles suggest a nuanced roles of fibroblast subtypes in either resolving or sustaining inflammation. Though traditionally seen as non-immune, pulp fibroblasts can produce complement proteins and modulate immune activity (9, 31). Our findings of distinct fibroblast populations in pulpitis versus healthy pulp suggest two, possibly overlapping, scenarios: fibroblasts may migrate into the inflamed pulp chamber from the root canal or from the periodontium, or, similar to macrophages, they may undergo functional shifts to help regulate the immune response.

In support for the first scenario of fibroblast migration, our data reveal fibroblast heterogeneity between healthy and inflamed samples. In healthy pulp (#HL1), resident fibroblasts predominantly expressed CXCL14, PTN, and CRABP1. In contrast, inflamed pulps – particularly #IP1 – contained infiltrating subsets resembling periodontal ligament (PDL) fibroblasts and fibroblasts from the apical papilla (AP), characterized by lower CXCL14 and higher CXCR4 expression. Notably, fibroblasts from AP appear to drive TLR4-mediated inflammation. CXCR4 has been reported to chemoattracts stem cells from the apical papilla (SCAP) to transmigrate into the root canal space as an endogenous cell source for pulp regeneration (32). These infiltrating fibroblasts, regardless of their labeling, likely migrate into the pulp to regulate inflammation and promote repair (9, 31) or indicate potential for pulp regeneration (32). Importantly, these fibroblast subsets also express immune-related genes, including complement factors and CXCL13, further blurring the classical immune versus stromal categorization.

In that context of cells misnomers, it should not slip our minds that cells have traditionally been labelled according to their shape (e.g., stellate reticulum or bacterial cells) or their location (e.g., pericytes or endothelial cells) without reflecting the distinct functions of the different cells that appear identical or anatomically colocalized under the light microscope. We expect similar studies employing Single-cell and spatial transcriptomic technologies to set free the fibroblasts (among other cell types) from this ambiguous category of names by reclassifying them according to their unique transcriptional profiles hence their precise role in tissue immunity and homeostasis (31).

Surprisingly and defying our hypothesis, our data show no significant differences in pro- and anti-inflammatory macrophage markers between samples diagnosed as RP and IP (Fig. 4F & G). For example, IL-1β, among other potent proinflammatory cytokine, was only mildly expressed in #IP1. These results challenge the notion that macrophages are the main drivers of inflammation resolution in pulpitis (22, 33). Instead, and moving beyond the traditional M1/M2 model, our findings suggest that the immune response is shaped by dynamic interactions between fibroblasts and immune cells, highlighting the plasticity of both populations in balancing inflammation and repair (34).

The spatial analysis comparing the region facing the carious lesion with the whole pulp chamber revealed distinct spatial inflammatory patterns. Consistent with previous studies linking caries depth to inflammatory mediator levels, these patterns appear driven by microbial endotoxins and localized cytokine activation (35). Notably, CD274 (PD-L1) was expressed only near the caries front in #IP1, suggesting an immune checkpoint response triggered by bacterial endotoxins (20). This is the first report, to our knowledge, showing localized CD274 overexpression in pulp tissue, highlighting its potential as a target for modulating pulpal inflammation. Inflammation near the caries front, though typically attributed to bacterial endotoxins, also involved complex spatial signaling. For instance, MS4A1 (CD20) and ITGAX (CD11c) were enriched in #IP2, indicating local B cell and dendritic cell activation. These signals, though weaker, were also present in RP, emphasizing the role of transcriptional context and immune cell distribution over individual marker expression levels. This underscores the translational potential of spatial transcriptomics to enable site-specific analysis of inflammation, highlighting significant interregional heterogeneity within the same pulp tissue which is a challenge for traditional diagnostic classifications (3, 4).

This study, the first to report spatial transcriptomic profiling of dental pulpitis, validates the potential of the technology to identify molecular signatures predictive of inflammation resolution versus progression. Despite the limitation of a small sample size, our findings confirm that some cases clinically diagnosed as irreversible pulpitis exhibit molecular signs of reversibility and may therefore be suitable candidates for conservative treatment. As we continue recruiting patients to validate our findings, we expect spatial transcriptomics to help uncover patterns of immune-fibroblast crosstalk with markers from both cell types that can distinguish RP from IP.

Dental pulpitis provides a uniquely accessible model for showing how molecular reclassification of disease can uncover hidden biological reversibility in conditions traditionally deemed irreversible. In doing so, this work establishes a blueprint for applying – well beyond dentistry – spatially resolved molecular diagnostics across inflammatory and degenerative diseases, advancing the broader fields of tissue preservation, precision diagnostics and personalized medicine.

In summary, spatial transcriptomics offers a powerful framework to reclassify pulpitis by its molecular signatures, advancing molecularly guided, pulp-preserving endodontic care to sustain tooth vitality for a lifelong function.

## Data Availability

The datasets generated and analysed during the current study will be available on the NCBI Gene Expression Omnibus (GEO) as soon as the manuscript is accepted.
